# Prefrontal cortex and cognitive control: new insights from human electrophysiology

**DOI:** 10.12688/f1000research.20044.1

**Published:** 2019-09-27

**Authors:** Alik S. Widge, Sarah R. Heilbronner, Benjamin Y. Hayden

**Affiliations:** 1 Department of Psychiatry, University of Minnesota, 3001 6th St SE, Minneapolis, MN, 55455, USA; 2 Department of Neuroscience, Center for Magnetic Resonance Research, and Center for Neuroengineering, University of Minnesota, 2021 6th St SE, Minneapolis, MN, 55455, USA

**Keywords:** Cognitive control, Electrophysiology, Conflict, cingulate, Local field potential

## Abstract

Cognitive control, the ability to regulate one’s cognition and actions on the basis of over-riding goals, is impaired in many psychiatric conditions. Although control requires the coordinated function of several prefrontal cortical regions, it has been challenging to determine how they work together, in part because doing so requires simultaneous recordings from multiple regions. Here, we provide a précis of cognitive control and describe the beneficial consequences of recent advances in neurosurgical practice that make large-scale prefrontal cortical network recordings possible in humans. Such recordings implicate inter-regional theta (5–8 Hz) local field potential (LFP) synchrony as a key element in cognitive control. Major open questions include how theta might influence other oscillations within these networks, the precise timing of information flow between these regions, and how perturbations such as brain stimulation might demonstrate the causal role of LFP phenomena. We propose that an increased focus on human electrophysiology is essential for an understanding of the neural basis of cognitive control.

## Cognitive control and psychiatry

Cognitive control refers to the ability to regulate one’s own cognitive activity and the actions driven by that activity in the presence of overarching goals ^1– 3^. It is especially important when faced with the need to withhold a response or to countermand a planned thought or action. For example, anyone who has lived with a toddler knows that “inside voice” is different from “outside voice”. The parent needs to teach the child that even something as basic as speaking volume must be linked to a seemingly arbitrary context and that a habit of speaking loudly must be controlled in some circumstances. A more clinically relevant example would come from touching a doorknob in a public building. That action may trigger thoughts of germs and may motivate hand-washing. But a patient with obsessive-compulsive disorder (OCD) undergoing exposure response prevention therapy may choose to deliberately delay hand-washing because of a higher-order goal of practicing not responding to obsessions.

Cognitive control involves three major components ^4– 6^. First, we must *maintain* an ever-changing internal mental representation of our long-term goals. Then, we must *monitor* our interactions with the world and compare the results of those interactions (or the likely results of intended interactions) with our goals and assess how closely they match. Finally, we must *adjust* our behavior, if need be, to better fit our goals. In other words, our brains function, in an abstract sense, like a closed-loop engineering control system.

A critical element of most control systems is an internal representation of the task and its contingencies. Detection of failure to achieve goals requires a representation of what those goals are; adjustment requires a judgment about which way the adjustment should go. Thus, an important part of control, which we do not focus on here, is the map of task state. There is some evidence that the orbitofrontal cortex (OFC) may be specialized for maintaining this type of representation ^7– 9^. Another possibility is that maps of state space are more widespread, perhaps in different forms. Such representations, relevant for control, may be stored adjacent to representations needed for the relevant processing (for example, in dorsal anterior cingulate cortex [dACC] ^10^).

Contexts that elicit control, such as conflict, can have two types of consequences. First, we can try to change course online (that is, adjust our immediate plans so that we do something different) ^11– 15^. Second, we may change strategies on subsequent trials or encounters. Over short time frames, this process is known as adjustment ^16, 17^. Over longer time frames, it is known as learning. Both processes are united in that they use monitoring signals to alter stimulus-output mappings. Together, these two forms of adjustment are often known as proactive and reactive control.

Notably, cognitive control overlaps quite a bit with the concept of self-control ^3, 18– 21^. Self-control, however, is slightly different. Specifically, it can be defined as the deliberate selection of an abstemious option that produces greater long-term benefits when faced with a more tempting option. Thus, it is a cognitive operation that generally requires cognitive control. As the study of one is likely to help shed light on the other, their differences must be kept in mind.

## Limits of animal models

Neurophysiological models of cognitive control have been difficult to fully test. For example, major open questions include how theta might influence other oscillations within these networks, the precise timing of information flow between these regions, and how perturbations such as brain stimulation might demonstrate the causal role of local field potential (LFP) phenomena. One major challenge in answering questions like these is the disconnect between results from human and non-human studies. Structurally, we do not know the homology between prefrontal regions in rodent, monkey, and human ^22– 24^. Most notably, although monkeys appear to have a region homologous to human dACC, the specifics are hotly debated ^10, 25^. In rodents, portions of dACC may be quite different, and there is likely no homologue of dorsolateral prefrontal cortex (dlPFC) ^23, 26^. Functionally, the overlaps are unclear as well. For example, dACC is frequently activated in human neuroimaging studies of conflict and associated control-related adjustment. On the other hand, primate neurophysiology studies, which have high temporal and spatial resolution, have generally failed to find single-unit correlates of cognitive conflict in this region ^27– 30^. The reasons for the disconnect between non-human primate electrode recording studies and human neuroimaging are not clear ^31^, although the lack of naturalness of the primate tasks may be a contributing factor ^32^.

This does not mean that non-human animal models are worthless. Indeed, animal models have proven quite useful in understanding the neural basis of, for example, response competition ^33– 36^, rule switching ^37– 40^, response inhibition ^41^, persistence ^42– 44^, and outcome monitoring ^45– 47^. These successes, indeed, are foundational in the field of cognitive control. Nonetheless, they do not provide a complete picture of cognitive control and they have some limitations. For example, we lack a clear and non-controversial model of cognitive conflict and the types of control needed for rapid learning, for control related to language, for culture, or arguably for self-control ^48^.

Consequently, humans are the most important model organism for the study of cognitive control. Unfortunately, the spatial resolution of electro-encephalography (EEG), the most prevalent human electrophysiological technique, may not be conducive to refining the specific functional roles of spatially neighboring PFC regions. It also may not pick up key nodes of the proposed network (for example, OFC ^49^). Magnetoencephalography, while offering potentially higher resolution, is still far from desired. Finally, functional magnetic resonance imaging, although it is the most widely used method, lacks sufficient spatial and temporal resolution to answer several key questions, such as those related to theta (see below).

**Figure 1.**
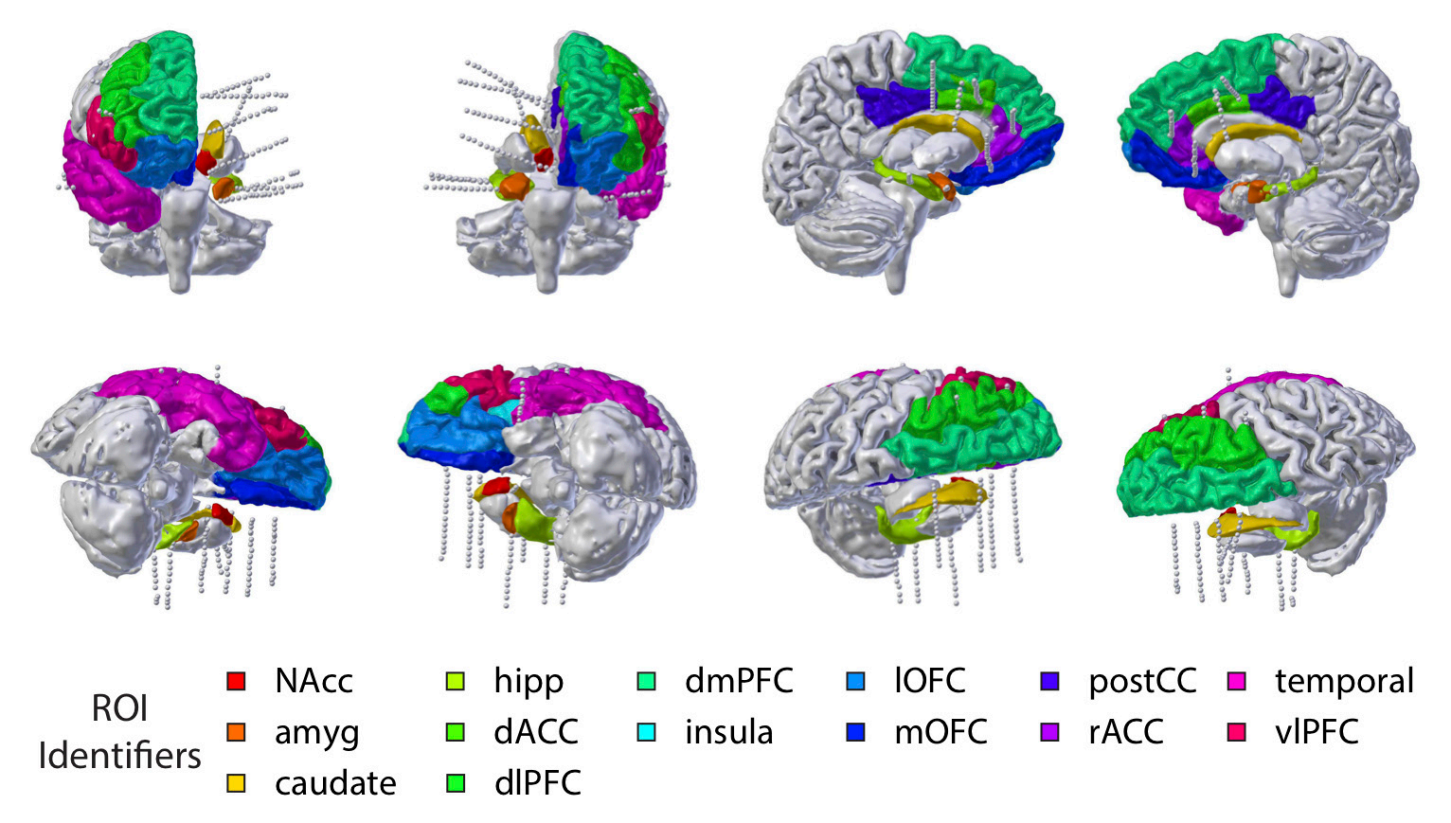
An example montage from a human intracranial subject. dACC, dorsal anterior cingulate cortex; dlPFC, dorsolateral prefrontal cortex; dmPFC, dorsomedial prefrontal cortex; lOFC, lateral orbitofrontal cortex; mOFC, medial orbitofrontal cortex; NAcc, nucleus accumbens; postCC, posterior cingulate; rACC, rostral anterior cingulate cortex; ROI, region of interest; vlPFC, ventrolateral prefrontal cortex.

Recent advances in neurosurgery offer the promise of intracranial recordings in humans. Patients with medication-resistant epilepsy often obtain relief from neurosurgery, wherein a seizure-originating focus is removed from their brains. Localizing an individual patient’s focus can require the implantation of temporary monitoring electrodes that effectively triangulate the seizure origin through monitoring of the LFP. Changes in patient and surgeon preference have driven rapid adoption of a specific monitoring technique called stereotactic EEG (stereo-EEG for short), where the monitoring electrodes are long cylindrical shanks placed through very small drill holes in the skull. These electrodes pass through superficial cortex and terminate in the deep brain, often close to the midline ( Figure 1). Thus, these patients may have continuous recordings from multiple PFC components for a period of 1 to 2 weeks. During this time, patients are typically resting in a hospital room, awaiting a seizure event, and are often able to perform psychophysical tasks. These task runs offer a unique opportunity to study cognitive control processes directly in humans. Next, we summarize the relationship between mental illness and cognitive control and then describe some of the benefits of intracranial studies.

## The transdiagnostic nature of cognitive control

Deficits in cognitive control have been demonstrated in almost every psychiatric illness (for example, 50– 57). For example, one hallmark of cognitive control is the ability to readily disengage from one train of thought or pattern of action to pursue an alternative. That alternative may be goal-aligned; thus, failure to disengage is often opposed to goals. People with OCD can have trouble disengaging from persistent anxious thoughts and the resulting rituals, people with post-traumatic stress disorder can have trouble disengaging from avoidance behavior driven by fear memories, and people with substance addiction can have trouble disengaging from cravings and the resulting drug-taking. In other words, cognitive control failures are a *transdiagnostic* phenomenon. Furthermore, such deficits can translate to controlled laboratory tasks of goal-aligned disengagement, such as reversal learning and cognitive conflict tasks ^58, 59^.

Disorders associated with cognitive control deficits are treatable through cognitive-behavioral therapy (CBT), which seeks to teach patients alternate (more adaptive) behaviors to replace their pre-potent or automatic responses. This could be thought of as providing a second automatic response that, once practiced, is somehow “easy” to select. The success of CBT suggests that control may be a target for remediation and disease treatment. Indeed, both invasive ^60^ and non-invasive ^61^ brain stimulation can improve performance on cognitive conflict tasks, the most common laboratory measure of cognitive control. That improvement is associated with changes in neurophysiological signatures of control.

It nevertheless remains difficult to use cognitive control directly as a diagnostic or therapeutic target. Psychiatric disorders are internally heterogeneous ^62, 63^. Control deficits might be more common among patients with a given diagnosis, but it does not follow that *every* individual with that diagnosis is guaranteed to have a control deficit. This may explain why all the effects just cited are themselves heterogeneous; for every disorder, there are also studies failing to find cognitive control deficits in a particular patient sample ^64^. Although this may sound like a case for pessimism, we believe it indicates the potential benefit that can accrue from more fine-grained and precise measures of cognitive control.

Given that control deficits are found across disorders, but heterogeneously, a logical approach would be to identify a test that detects such deficits by studying a large sample of patients with a variety of diagnoses. Two recent studies took the first steps on that road. One applied a comprehensive neurocognitive battery and a variety of psychophysical tasks to 420 patients across mood and anxiety diagnoses, identifying six subgroups through a data-driven clustering approach ^65^. These subgroups could be discriminated by their performance on a go/no-go task when that performance was expressed relative to age- and sex-corrected healthy volunteer performance. A different approach used the two-step learning paradigm ^66^, a laboratory task in which subjects must navigate a decision tree to find the most rewarding outcomes. This paradigm has the advantage of a well-accepted parametric behavior modeling approach ^67^, which, when applied to an individual, yields a single number quantifying capacity for “model-based” control processes. Gillan *et al*. developed a population norm for this parameter in 1,413 online subjects, establishing a means to quantify individual-level control deficits ^66^. The authors then showed that subjects with these deficits were more likely to report psychiatric symptoms, particularly those related to perseveration and compulsion.

## The anatomic basis of cognitive control

A brief detour into the neuroanatomy of cognitive control reveals a clear overlap between regions associated with control and those associated with psychiatric disease. Psychiatric disorders involve abnormalities in many brain regions, but the most consistent of these are in the prefrontal cortico-basal ganglia network ^68^. Similarly, cognitive control is closely associated with the prefrontal cortex, so much so that it is sometimes thought to be the defining feature of this large and heterogeneous region ^1^. This is, of course, overly simplistic, but it is clear that many prefrontal regions are closely involved in cognitive control. Most prominent among these are the dACC and dlPFC. Both neuroimaging and electrophysiological studies, from humans and non-human animals, indicate that these two regions are centrally involved in cognitive control ^4, 10, 25^. In addition, lesions or damage to these regions tend to produce impairments in control. Other regions that are associated with cognitive control are the ventrolateral prefrontal cortex ^69^, the dorsomedial PFC (DMPFC) ^70^, and the OFC ^71, 72^. Some work even implicates the striatum, although its role is far from clear ^38, 54, 73, 74^.

Models of prefrontal function ascribe to these regions several features that are critical to cognitive control: (1) they participate in *monitoring* goal-relevant variables, (2) their activity reflects the evaluation of costs and benefits of choice alternatives, (3) they *maintain* rule sets and goal-related information in working memory, and (4) they *adjust* adaptive biases toward more successful behavior. The evidence linking prefrontal cortex to each of these functions is strong. However, the specific assignment of regions to functions remains unclear; indeed, some recent theories hold that it may be impossible to link function to region in a one-to-one manner. Instead, regions may be better described as existing on a hierarchy of control ^75– 78^.

More formally, cognitive control can be conceptualized as a neural instantiation of ideas from engineering: monitor and controller. These terms originate in engineering control theory and refer to elements of a control system that detect the need for control and implement it, respectively. By this same logic, we may hypothesize that OFC serves as a source of reward information that drives control decisions and that DMPFC serves as a final gateway to motor systems. This speculative assignment of roles to regions offers a suggestion for why dACC appears to be so central: it functions as a hub point for the initiation of control ^79– 81^. This theory has the potential to integrate several seemingly disparate findings about dACC, including its role in monitoring reward outcomes, its role in choice processes, and its spatial reference frames, especially the frame of actions ^82– 88^.

## Local field potential synchrony may bind frontal regions to achieve control

**Figure 2.**
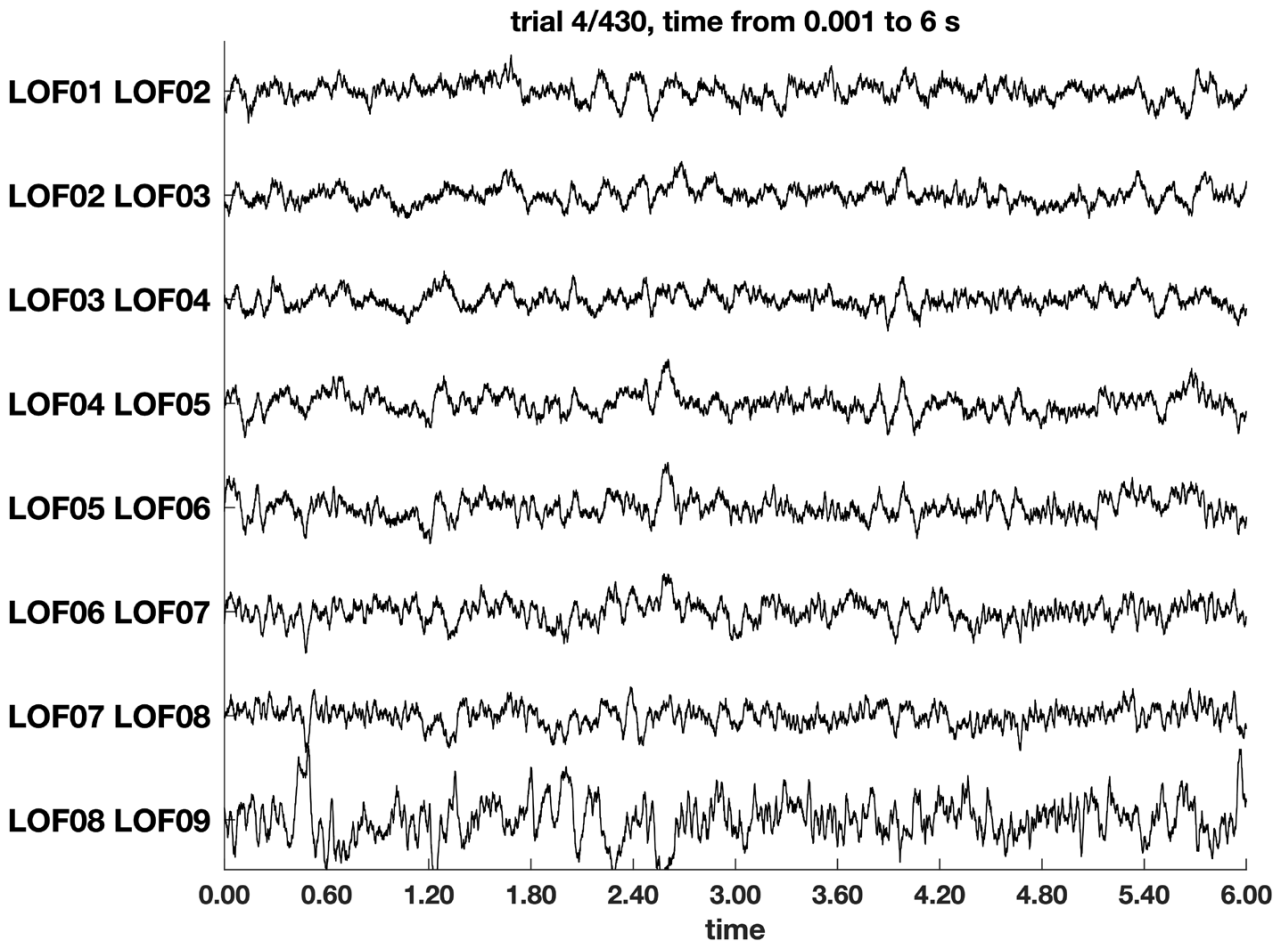
Example of stereotactic electro-encephalogram recordings—in this case, eight bipolar-referenced channels from the left orbitofrontal cortex (LOF)—during one trial of a cognitive control task. Example of stereotactic electro-encephalogram recordings—in this case, eight bipolar-referenced channels from the left orbitofrontal cortex (LOF)—during one trial of a cognitive control task.

The broad involvement of dACC, dlPFC, and other prefrontal regions highlights the fact that cognitive control is a network function. This raises a question: how are top-down control signals and bottom-up signals of the need for control routed? Recent work suggests that networks of cognitive control can be established through cross-regional synchronization of oscillations in the LFP. (Some example traces of real patient data are shown in Figure 2). The general notion that LFP synchrony is a mechanism for brain communication is nearly 15 years old ^89^ and the theory continues to be refined ^90– 93^. This includes a continued expansion of the definition of “synchrony”, to encompass spike-field coherence ^90, 94^ measures related purely to the phase of the LFP oscillation, or as coupling between a lower-frequency and higher-frequency oscillation ^91, 95– 97^. Furthermore, evidence continues to build that LFP synchrony plays a role in long-range brain areal communication. There is particularly strong evidence in sensation- and memory-related research ^98, 99^.

Oscillations in the theta frequency band (5–8 Hz) are particularly relevant for top-down communication ^100^. Theta rhythms in the scalp EEG recorded over PFC have long been associated with cognitive control, especially as studied in conflict paradigms ^55, 101– 103^. In theory, these power increases at the scalp might reflect changes in the synchrony of underlying cortical structures, where greater synchrony of neural firing leads to larger induced dipoles that are more easily observed at the scalp. If this is true, invasive neural recordings during similar tasks should show increases in theta phase synchrony (coherence or phase locking) that precede successful cognitive control.

## Human intracranial recording as a tool for studying control

Thus far, invasive human electrophysiology studies support the thesis that LFP synchrony is a key component in fronto-cingulate network formation and function across multiple components of cognitive control. As humans are asked to make decisions according to increasingly abstract rules (that is, to exercise goal-maintenance aspects of control), theta phase synchrony between PFC and motor regions increases ^91^. Directed analysis shows cross-frequency coupling between these same regions, wherein PFC’s phase drives the amplitude of high-frequency M1 oscillations. A parallel study extended this finding to control during cognitive conflict. After subjects receive error feedback during a cognitive conflict task (signaling an increased need to deploy control), there is directed information transfer from medial to lateral PFC electrodes ^104^. Furthermore, that information transfer (assessed by mutual information calculations) is strongest between 4 and 8 Hz, the canonical theta band. Using the same task, our group recently showed that this theta synchrony extends well beyond PFC ^105^. We showed that cognitive control tasks engage pairwise correlations between PFC, cingulate, and subcortical structures and that the majority of high-influence correlations were in the theta band ^105^. This may reflect a link between control and attention; PFC synchronizes with parietal cortex to entrain parietal oscillations to ongoing intermittent signals, increasing their chance of successful detection ^100^. The dlPFC-dACC control framework (described above) is also supported by intracranial electrophysiology. A recent study examined single-unit activity in humans during performance of a cognitive conflict task ^15^. As in macaques, there were only modest neuronal firing rate correlates of conflict in dACC and negligible ones in dlPFC. However, conflict substantially altered spike-phase coherence in dACC and spike-field coherence in dlPFC. The much stronger effects in the LFP domain than in the spiking domain were striking, especially given the failures to find neural correlates of conflict at the unit level in past studies. The authors proposed that the single units may serve as soloists that drive a larger oscillatory choir that in turn drives behavior ^15^. An open question is whether these networks can be perturbed to demonstrate causality between LFP synchrony and successful control.

Human experimental opportunities are an excellent opportunity for causal testing of cognitive control theories because specific brain regions’ function can be perturbed through electrical stimulation. Although seizures are always a concern in subjects with known epilepsy, many groups have conducted intermittent stimulation experiments in these populations. In those studies, there were no major adverse events and there were several reports of augmented function ^106– 109^. Arguably, the first report of such an experiment applied to cognitive control was a pair of patients in whom a subjective sense of “the will to persevere” could be evoked through rostral ACC stimulation ^110^. That study did not quantify the potential control change using a behavioral task, but another recent study did. In psychiatric patients with deep brain stimulation electrodes in the internal capsule, we increased the power of theta rhythms in both lateral and medial PFC and, by doing so, improved subjects’ performance on the multi-source interference task ^60^. The next step would be defining stimulation patterns that can specifically alter coherence, cross-frequency coupling, or other synchrony metrics. This may be possible by locking stimulation to the phase of a band-limited oscillation, which has recently become possible with advances in real-time signal processing ^111– 113^.

## Conclusions

Cognitive control has long been understood to require the coordinated function of multiple PFC structures. Recent advances in the study of control have begun to dissect what each of these regions contributes, how they collectively detect the need for control, and how they then bias lower-level cognitive processes to achieve a desired result. Access to human intracranial recording sites has allowed great advances in this domain. Specifically, it has allowed confirmation and extension of important theories about the neural basis of control in the organism of interest (humans) rather than in model organisms. This is important because of possible dishomology between human and model organism structure and function in key regions and because of doubt about our ability to elicit human-like control in model organisms. A key next step is linking this network-level recording to formal theories of cognitive control ^3, 6^. Specific aspects of theoretical/computational models should load onto sub-regions of PFC but this assumption has not yet been tested. Furthermore, if the theta synchrony theory is correct, it should be possible to track sequential flow of a computation through PFC and ACC, for example, through spectrally resolved directed connectivity metrics.

Relatedly, human intracranial opportunities allow direct manipulation of these circuits in the human brain. For instance, as described above, we have verified a role of cortico-striatal circuits in control by manipulating those circuits with deep brain stimulation. Evidence from other cognitive systems suggests that the inter-regional communication necessary for top-down control may require synchronized LFP oscillations. Different groups have found evidence for phase-related measures (for example, coherence), phase-amplitude coupling, and amplitude-amplitude measures (mutual information) as measures of that synchrony. Overall, this hypothesis is plausible but incompletely tested. With rapid advances in recording and stimulation capabilities, particularly for experiments with human volunteers, that will soon change. Furthermore, these experiments often use electrical stimulation to perturb networks of cognitive control, increasingly in activity-dependent closed-loop paradigms. Those approaches offer the prospect not only that we will understand control better in years to come but that such studies will lead directly to new therapies targeting deficits in control.
